# Performance of the cobas EBV and cobas BKV assays: multi-site comparison of standardized quantitation

**DOI:** 10.1128/jcm.00267-24

**Published:** 2024-07-24

**Authors:** Laura Mannonen, Pia Jokela, Marianne Kragh Thomsen, Sabine Yerly, Gustavo Cilla, Daniel Jarem, Jesse A. Canchola, Mark Hopkins

**Affiliations:** 1 Department of Clinical Microbiology, HUS Diagnostic Center, HUSLAB, Clinical Microbiology, University of Helsinki and Helsinki University Hospital, Helsinki, Finland; 2 Department of Clinical Microbiology, Aarhus University Hospital, Aarhus, Denmark; 3 Laboratory of Virology, Diagnostic Department, Geneva University Hospitals, Geneva, Switzerland; 4 Department of Microbiology, Donostia University Hospital and Biodonostia Health Research Institute, San Sebastián, Spain; 5 Clinical Development and Medical Affairs, Roche Molecular Systems, Inc., Pleasanton, California, USA; 6 CDMA Biometrics, Biostatistics Group, Roche Molecular Systems, Inc., Pleasanton, California, USA; 7 Department of Virology, Barts Health NHS Trust, London, United Kingdom; 8 Department of Infection and Immunity, Liverpool University Hospitals NHS Trust, Liverpool, United Kingdom; Mayo Clinic Minnesota, Rochester, Minnesota, USA

**Keywords:** Epstein-Barr virus, BKV, post-transplant monitoring, laboratory-developed test, assay standardization, viral load quantitation, assay variability, assay performance

## Abstract

Guidelines recommend monitoring of Epstein-Barr virus (EBV) and BK virus (BKV) in solid organ and hematopoietic stem cell transplant patients. The majority of quantitative DNA testing for EBV and BKV employs unstandardized individual laboratory-developed testing solutions (LDTs), with implications for accuracy, reproducibility, and comparability between laboratories. The performance of the cobas EBV and cobas BKV assays was assessed across five laboratories, using the World Health Organization International Standards (WHO IS) for EBV and BKV, and the National Institute of Standards and Technology Quantitative Standard for BKV, and results were compared with the LDTs in use at the time. Methods were also compared using locally sourced clinical specimens. Variation was high when laboratories reported EBV or BKV DNA values using LDTs, where quantitative values were observed to differ by up to 1.5 log_10_ unit/mL between sites. Conversely, results from the cobas EBV and cobas BKV assays were accurate and reproducible across sites and on different testing days. Adjustment of LDTs using the international standards led to closer alignment between the assays; however, day-to-day reproducibility of LDTs remained high. In addition, BKV continued to show bias, indicating challenges with the commutability of the BKV International Standard. The cobas EBV and cobas BKV assays are automated, aligned to the WHO IS, and have the potential to reduce the variability in viral load testing introduced by differences in LDTs. Standardization of reporting values may eventually allow different centers to compare data to allow clinical decision thresholds to be established supporting improvements in patient management.

**IMPORTANCE:**

The application of center-specific cut-offs for clinical decisions and the variability of LDTs often hinder interpretation; thus, the findings reported here support the need for standardization in the field of post-transplant monitoring of EBV and BKV to improve patient management. Alongside the choice of assay, it is also important to consider which standard to use when deciding upon a testing methodology. This is a call to action for standardization, as treatment for EBV and BKV is driven by viral load test results, and the more accurate and comparable the test results are across institutions, the more informed and better the treatment decisions can be.

## INTRODUCTION

Transplant patients have depressed immune function as a result of immunosuppressive medication to prevent graft rejection, putting them at increased risk for viral and bacterial infections (1). Two potential viral infections of concern in transplant recipients are Epstein-Barr virus (EBV) infection and BK virus (BKV) infection.

EBV infection is widespread, with most people having non-specific or asymptomatic primary infection before the age of 10 years, and latent infection in 90% of adults (2, 3). EBV causes infectious mononucleosis in primarily infected adolescents and young adults (3) and is associated with several types of cancer, including nasopharyngeal carcinoma, Burkitt lymphoma, and Hodgkin lymphoma (2). EBV can cause lymphoproliferative disorders in individuals with congenital or acquired immunodeficiency, including transplant recipients (2). Post-transplant lymphoproliferative disorder (PTLD) is a severe post-transplant complication of EBV infection, which can occur particularly when transplants to EBV-negative recipients are performed from EBV-positive donors (4–6). Given the risks associated with EBV infection in transplant patients, determination of the EBV serostatus of the recipient and donor prior to transplantation by detection of antibodies to the EBV capsid antigen is important; however, these measurements are not useful once immunosuppressive therapy has started (4, 7). Guidelines also recommend that EBV load is closely monitored in patients at risk of PTLD (e.g., especially in EBV-seronegative recipients at the time of transplant) with adjustment of immunosuppression based on viral load (4, 8–10). As a reference, ECIL-6 EBV in hematopoietic stem cell transplantation (HSCT) guidelines state some authors employ thresholds between 1,000 and 40,000 copies/mL depending on the sample type but admit there is no consensus, as such, often the rate of increase is used in which case reproducibility is crucial (10).

Seropositivity for BKV is high in the general population and infection rarely causes significant consequences in immunocompetent individuals (11). However, in immunocompromised hosts, primary infection with, or reactivation of, BKV can lead to serious complications, including BKV-associated nephropathy, irreversible tissue damage and graft failure in renal transplant patients, and BKV-associated hemorrhagic cystitis in HSCT patients, together with rare complications including pneumonia, encephalitis, and retinitis (11–13). Unlike other transplant-associated infections such as cytomegalovirus infection, donors and recipients are not screened for BKV serostatus prior to transplant; however, there is increasing evidence to suggest this would be useful (14). Guidelines currently recommend monitoring of BKV DNA levels post-renal transplantation, with adjustment of immunosuppressive regimens based on BKV levels (9, 13, 15, 16), and post-HSCT (17). For reference, recommendations vary, with ECIL (17) hemorrhagic cystitis in HSCT referring to plasma values >1,000 copies/mL and renal association clinical practice guidelines in post-operative care in the kidney transplant recipient referring to values >10,000 copies/mL (16).

However, the effectiveness of both EBV and BKV load monitoring has been called into question due to the variability in viral load testing (4, 18). Interassay and interlaboratory variation in quantitative results have implications for patient management where viral load can influence management strategies, in particular when different laboratories report inaccurate or discordant results (18). As well as the use of different standards, viral genotypic variation may also contribute to variability in quantitative testing (8, 14). Despite improvements in some viral assays with the use of World Health Organization International Standards (WHO IS), a high degree of interlaboratory variability has still been reported (19). There is therefore a need for reliable, standardized EBV and BKV DNA quantification.

The cobas EBV and cobas BKV assays for use on the cobas 6800/8800 systems (20) are real-time PCR-based tests with dual-target technology for direct detection and quantification of EBV or BKV DNA to aid in the diagnosis and management of EBV and BKV infections in solid organ transplant patients and HSCT patients. The objectives of the study were to assess the analytical performance (linearity, accuracy, and reproducibility) of the cobas EBV and cobas BKV assays (Roche Molecular Systems, Pleasanton, CA, USA), using the respective WHO IS or National Institute of Standards and Technology (NIST) standard, compared with the LDTs in use at each testing site; and to evaluate assay performance using locally sourced clinical specimens by method comparisons between the cobas EBV or cobas BKV assays and LDTs.

## MATERIALS AND METHODS

### Study design

This was a multicenter evaluation with analytical and clinical assessments performed across five sites in Europe. Details of the assays are described in Tables 1 and 2. Invalid results were excluded from the analysis.

**TABLE 1:** Details of EBV LDT at each testing site^*a*^

	Extraction		Amplification		Values (copies/mL)			Calculated conversion factor (IU/cp)	Standardized values (IU/mL)		
Site	Assay	Instrument	Assay (manufacturer)—Target	Instrument	Lower (LLoQ)	Upper (ULoQ)	Cut-off for TND^*b*^		Lower (LLoQ)	Upper (ULoQ)	Cut-off for TND
*LDTs*											
**1**	QIAsymphony DSP Virus/Pathogen Midi Kit (Qiagen)	QIAsymphony (Qiagen)	PCR EBV QS-RGQ (Artus—Qiagen)—EBNA-1 (97 bp)	ViiA 7 Real-Time PCR System (Thermofisher Scientific)	316 (manufacturer-defined)	1.00 × 10^7^ (manufacturer-defined)	500 (laboratory-defined)	0.12	37.9	1.20 × 10^6^	60.0
**2**	QIAsymphony Virus DSP (Qiagen)	QIASymphony (Qiagen)	LDT—EBNA-LP	Rotorgene (Qiagen)	250 (laboratory-defined)	1.56 × 10^6^ (laboratory-defined)	Not defined	0.19	47.5	2.96 × 10^5^	Not defined
**3**	DNA/Viral NA SV Kit (Roche Diagnostics)	MagNA Pure 96 (Roche Diagnostics)	LDT—BALF5	ABI7500 or ABI7900 (Applied Biosystems)	500 (laboratory-defined)	5.00 × 10^6^ (laboratory-defined)	Not defined	0.34	170	1.70 × 10^6^	Not defined
**4**	NUCLISENS Emag (BioMérieux)	eMAG (BioMérieux)	LightCycler EBV Quant Kit (Roche Diagnostics)—EBNA (166 bp)	LightCycler 2.0 (Roche Diagnostics)	1.0 × 10^3^ (manufacturer-defined)	2.0 × 10^7^ (manufacturer-defined)	229 (manufacturer-defined)	4.2	4.2 × 10^3^	8.4 × 10^7^	961.8
**5**	MagNa Pure Compact Nucleic Acid Isolation kit (Roche Diagnostics)	MagNa Pure Compact (Roche Diagnostics)	LDT—BNRF1 (74 bp)	LightCycler 480 System (Roche Diagnostics)	Reports in IU/mL			N/A	100 (laboratory-defined)	1.00 × 10^6^ (laboratory-defined)	25 (laboratory-defined)
*Comparator*											
			cobas EBV assay (Roche Diagnostics)—EBNA-1; BMRF	cobas 6800/8800 Systems (Roche Diagnostics)	Reports in IU/mL			N/A	35.0 (manufacturer-defined)	1.00 × 10^8^ (manufacturer-defined)	18.8 (manufacturer-defined)

^*a*^ Cp, copies; EBV, Epstein-Barr virus; IU, international units; LDT, laboratory-developed testing solutions; LLoQ, lower limit of quantitation; N/A, not applicable; PCR, polymerase chain reaction; TND, target not detected; ULoQ, upper limit of quantitation.

^*b*^ This is the lower limit of detection, either as reported by the manufacturer or internally validated by the institution.

**TABLE 2:** Details of BKV LDT at each testing site^*a*^

	Extraction		Amplification		Values (copies/mL)			Calculated conversion factor (IU/cp)	Standardized values (IU/mL)		
Site	Assay	Instrument	Assay (manufacturer)—Target	Instrument	Lower (LLoQ)	Upper (ULoQ)	Cut-off for TND^*b*^		Lower (LLoQ)	Upper (ULoQ)	Cut-off for TND
***LDTs***											
**1**	Protocol generic	eMag (BioMérieux)	PCR BK virus R-Gene (BioMérieux)—Small T Antigen	ViiA 7 Real-Time PCR System (Thermofisher Scientific)	316 (manufacturer-defined)	1.2 × 10^9^ (manufacturer-defined)	65 (manufacturer-defined)	1.8	569	2.16 × 10^9^	117
**2**	QIAsymphony Virus DSP (Qiagen)	QIAsymphony (Qiagen)	LDT—VP1 capsid	Rotorgene (Qiagen)	500 (laboratory-defined)	1.00 × 10^6^ (laboratory-defined)	Not defined	0.2	100	2.00 × 10^5^	Not defined
**3**	DNA/Viral NA SV Kit (Roche Diagnostics)	MagNA Pure 96 (Roche Diagnostics)	LDT—Large T-Antigen	ABI7500 or ABI7900 (Applied Biosystems)	400 (laboratory-defined)	4.00 × 10^9^ (laboratory-defined)	Not defined	1.6	640	6.40 × 10^9^	Not defined
**4**	NUCLISENS eMag (BioMérieux)	eMag (BioMérieux)	LightMix Kit Polyomaviruses JC and BK (TIB MOLBIOL, Roche Diagnostics)—Small T-Antigen (175 bp)	LightCycler 2.0 (Roche Diagnostics)	1.10 × 10^4^ (manufacturer-defined)	1.10 × 10^8^ (manufacturer-defined)	1.10 × 10^3^ (manufacturer-defined)	Due to the variability of results for the 1,000 IU/mL panel, a statistically reliable conversion factor could not be created			
**5**	MagNa Pure Compact Nucleic Acid Isolation kit (Roche Diagnostics)	MagNa Pure Compact (Roche Diagnostics)	PCR BK virus R-Gene (BioMérieux)— Small T-Antigen	LightCycler 480 (Roche Diagnostics)	300 (laboratory-defined)	1.00 × 10^11^ (laboratory-defined)	65 (laboratory-defined)	2.1	630	2.10 × 10^11^	137
*Comparator*											
			cobas EBV assay (Roche Diagnostics)—VP2; Small T-Antigen	cobas 6800/8800 Systems (Roche Diagnostics)	Reports in IU/mL			N/A	21.5 (manufacturer-defined)	1.00 × 10^8^ (manufacturer-defined)	21.5 IU/mL (manufacturer-defined)

^*a*^ cp, copies; BKV, BK virus; IU, international units; LDT, laboratory-developed testing solutions; LLoQ, lower limit of quantitation; N/A, not applicable; TND, target not detected; ULoQ, upper limit of quantitation.

^*b*^ This is the lower limit of detection, either as reported by the manufacturer or internally validated by the institution.

### Analytical panels

Analytical panels were prepared by diluting the WHO EBV IS (NIBSC code: 09/260) and the WHO BKV IS (NIBSC code: 14/212) to the following concentrations; negative; 1,000 IU/mL; 6,000 IU/mL; and 10,000 IU/mL. The NIST BKV DNA Quantitative Standard (NIST BKV) was diluted to the following concentrations: negative; 1,000 cp/mL; 6,000 cp/mL; and 10,000 cp/mL. Concentrations do not span the entire analytical measurement range, particularly the upper range, as the panels were intended to focus on reliability at relevant pre-emptive decision thresholds and the accuracy and reproducibility at the lower end of the range, with early detection of viral load changes from reactivation or primary infection highly relevant for EBV and BKV transplant monitoring. Samples were diluted using EBV-DNA-negative or BKV-DNA-negative human EDTA-plasma (confirmed as negative using the cobas assays and the local LDT), with a fill volume of 1.2 mL per replicate. The panels were anonymized (concentration not listed on panel), stored at −80°C, and shipped to the study sites on dry ice. At each site, three replicates per concentration were tested over three different days on the cobas 6800/8800 systems and the LDT (total of 18 replicates per level) to evaluate reproducibility. Panel materials were single-use vials and were shipped frozen, thawed, and tested on both platforms at the same time, without additional freeze/thaw cycles. Invalid results were repeated where volume permitted additional testing.

### Clinical samples

Anonymized leftover clinical EDTA-plasma samples were retrospectively sourced from storage at five study sites where EBV/BKV DNA measurements were available from routine monitoring (minimum volume required 500 µL) and selected based on historical EBV/BKV viral load results determined by the LDTs in use at the site in three categories: target not detected (TND), detectable but not quantifiable [<LLoQ (lower limit of quanitation)], and quantifiable. The number of samples required for each category and target was based on statistical power and study feasibility, with each site contributing up to 140 tests in total across all three categories, where possible, and per target (EBV or BKV). This approach was based on CLSI EP09-A2 (21) for method comparisons, as well as considering a non-burdensome approach for each site to contribute to the study. Samples were collected between 08 March 2018 and 27 November 2019 and were frozen between testing on LDT, which was standard of care, and testing on the cobas EBV and BKV assays. Invalid results were repeated where volume permitted additional testing.

### Analysis

Statistical analyses were carried out using SAS/STAT software version 9.4 (22–25). All confidence intervals (CIs) were calculated using a 95% confidence level. The Studentized residuals method was used for outlier discovery. An outlier was determined if the absolute value of the Studentized residual was greater than 4. Linearity was assessed by comparing the mean log_10_ of the observed concentration with the predicted value from an ordinary least squares regression fitted across panel members. Accuracy was assessed by comparing the mean log_10_ of the observed concentration for each panel member and comparing it to the nominal concentration. Reproducibility was evaluated using a random effects model between sites and between days using the log_10_ transformed results. Standard deviation, log-normal percent coefficient of variation, and total log-normal coefficient of variation were calculated for each positive panel member. Method comparison of the LDTs at each site with the cobas EBV and cobas BKV assays was assessed using Deming linear regression analysis as well as ordinary least squares regression. The coefficient of determination, standard errors, and 95% CIs for the slopes and intercepts were calculated. Bias was assessed using Bland–Altman analysis. Method concordance was assessed across the detection range of the assays and at key breakpoints and any medically relevant decision points, such as pre-emptive treatment thresholds, to evaluate potential clinical implications of differences between the assays.

### Conversion factor

Analytical panel testing results, which used the WHO IS, were used to calculate a conversion factor to compare LDT results provided in cp/mL with the cobas EBV and cobas BKV assay results provided in IU/mL. The results from Methods 1 and 2 (described below) were appraised and if agreement was not acceptable, then a conversion factor was not applied. We employed two distinct conversion factor methods to ensure the robustness and reliability of our results, thereby enhancing the accuracy and confidence in the outcomes of our study.

#### Method 1: conversion factor calculation using a linear regression model

The following steps were used to estimate a conversion factor in IU/cp and cp/IU using the regression model described: (i) fit an ordinary least square (OLS) regression line within the linear range of the assay: Y=mX+b, where Y=log_10_ (observed in cp/mL), X=log_10_ (target concentration in IU/mL), m = slope, b = y-intercept; (ii) using the fitted OLS line from Step 1, calculate the predicted value for each of the three levels; (iii) average across the three predictions to get the IU/cp estimate; (iv) take the inverse of the value to generate the cp/IU.

#### Method 2: conversion factor calculation using the naïve method to buttress Method 1 results

The following steps were used to estimate a conversion factor in IU/cp and cp/IU using the naïve method: (i) calculate the mean log_10_ (observed quantitation in cp/mL) for each level; (ii) for each level, calculate the conversion factor as follows:


$$conversion\;factor\;(IU/cp)=target\;conc\;(IU/mL)/10^{\left[mean\;log10(observed\;quantitation\;in\;cp/mL)\right]}$$

(iii) average across the three conversion factor estimates to get the IU/cp estimate; and (iv) take the inverse of the value to generate the cp/IU.

#### Conversion factor—site-specific information

For site 5, the LDT results for EBV were already reported in IU/mL and thus no conversion was necessary. For site 4, conversion was not performed and the LDT results for BKV were reported in cp/mL only. The mean observed log difference for IU/mL vs copies/mL (bias) was calculated using the equation: average bias = linearized quant (log_10_ cp/mL) − target concentration (log_10_ IU/mL), where the log_10_ linearized quant is the predicted value from an equation fit on the data points in the dilution series with a slope of 1.0 (i.e., a constant value from top to bottom).

## RESULTS

The sample size breakdown for the analyzable samples tested is shown in Tables S1 and S2.

### Interlaboratory analytical performance using international standards

For the analytical performance study, a total of 515 samples were tested with the cobas EBV and cobas BKV assays (191 and 324 samples, respectively). Six samples were repeated for the cobas EBV assay and eight samples were repeated for the cobas BKV assay. In total, five invalid results were generated for the cobas EBV assay, total error rate of 2.6% (5 invalids of 191 samples run), and one invalid result for the cobas BKV assay, total error rate of 0.3% (1 invalid of 324 samples run), and were excluded from further analysis. The final analyzable sample sizes were 180 and 315 for EBV and BKV targets, respectively.

#### cobas EBV

**Fig 1:**
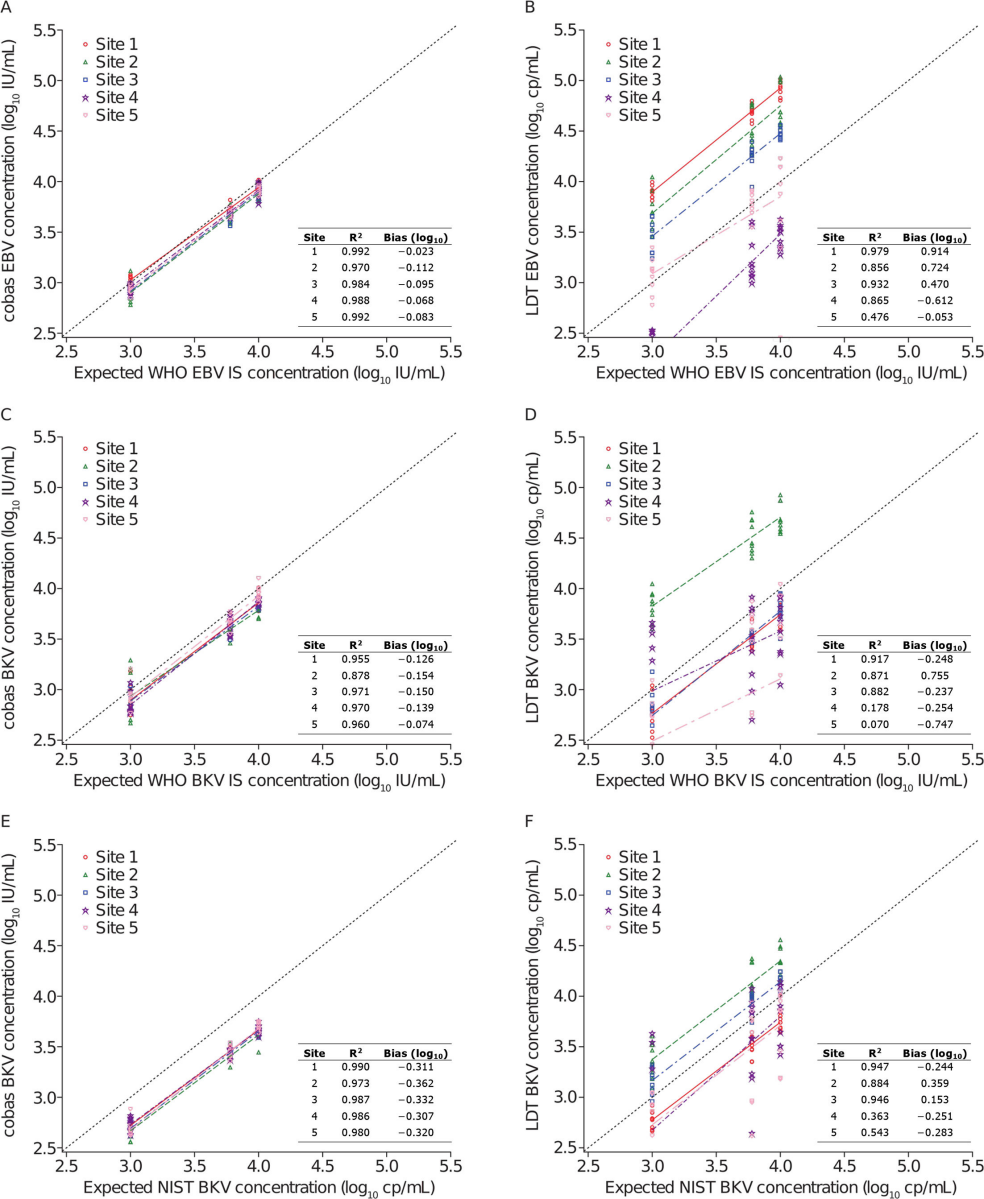
Analytical assay performance panels at each study site. Data points from each site are presented along with their color-coded regression lines. (**A**) cobas EBV concentration compared with the expected WHO EBV IS concentration; (**B**) LDT EBV concentration compared with the expected WHO EBV IS concentration;^*^ (**C**) cobas BKV concentration compared with the expected WHO BKV IS concentration; (**D**) LDT BKV concentration compared with the expected WHO BKV IS concentration; (**E**) cobas BKV concentration compared with the expected NIST BKV standard concentration; (**F**) LDT BKV concentration compared with the expected NIST BKV standard concentration. BKV, BK virus; cp, copies; EBV, Epstein-Barr virus; IU, international units; LDT, laboratory-developed testing solutions; NIST BKV, National Institute of Standards and Technology DNA quantitative standard for BKV; WHO BKV IS, WHO international standard for BKV; WHO EBV IS, WHO international standard for EBV. ^*^EBV LDT results for site 5 were reported in IU/mL.

Replicates of the WHO EBV IS at three dilution points were co-tested using both the cobas system and local LDTs at five European sites. Testing at all five study sites found that the cobas EBV panel produced results that consistently showed little variation from the expected values by dilution of the WHO EBV IS, with bias ranging from −0.023 to −0.112 log_10_ (Fig. 1A; Table S3). By contrast, the LDTs for each site produced varying results, with bias ranging from −0.053 to 0.914 log_10_ (Fig. 1B; Table S3).

The interlaboratory assessment showed a high agreement of cobas EBV DNA quantitation with the expected WHO EBV IS concentration across all five sites with cobas EBV linearity in the range of 0.970 to 0.992 (Table S3). There was significantly less agreement observed using the LDTs, where corresponding linearity values were in the range of 0.476 to 0.979 (Table S3).

**Fig 2:**
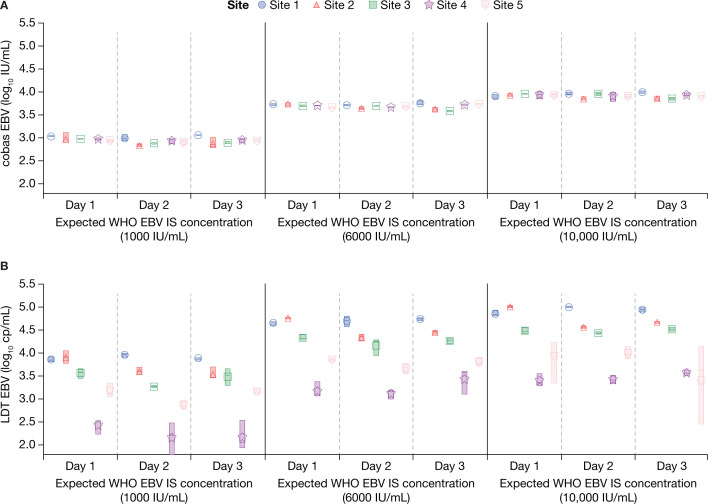
Reproducibility at each site across the three testing days at three different expected WHO EBV IS concentrations. (**A**) cobas EBV; (**B**) LDT EBV.^*^ cp, copies; EBV, Epstein-Barr virus; IU, international units; LDT, laboratory-developed testing solutions; WHO EBV IS, WHO international standard for EBV. ^*^EBV LDT results for site 5 were reported in IU/mL.

Similarly, the cobas EBV results were consistently reproducible across the three testing days (Fig. 2A), while the LDTs at each site, each with their designs and which were not standardized to the IS, produced variable results (Fig. 2B).

#### cobas BKV

Replicates of the WHO BKV IS and NIST BKV standards at three dilution points were similarly co-tested using both the cobas assays and local LDTs. The cobas BKV produced results that consistently showed little variation from the expected values when assessing the WHO BKV IS (Fig. 1C), whereas the LDT agreement with the WHO BKV IS was variable (Fig. 1D). However, when assessed with the NIST BKV standard, which is defined in copies/mL and not aligned to the WHO IS, the cobas BKV assay reported lower BKV values (Fig. 1E). The LDTs at sites 1, 4, and 5 appeared to detect in a similar range to the cobas BKV assay; the remaining LDTs detected higher than expected concentrations of BKV (Fig. 1F).

Interlaboratory assessment showed consistently high performance for BKV DNA quantitation across all five sites using the cobas BKV assay with BKV linearity and bias in the range 0.878 to 0.971 and −0.074 to −0.154 log_10_, respectively, for WHO BKV IS (Table S4). However, there was a poor agreement for BKV quantitation reported using LDT workflows and using the WHO BKV IS, where corresponding linearity and bias values were in the range of 0.070 to 0.917 and −0.747 to 0.755 log_10_, respectively. Quantitation of the NIST BKV standard also varied considerably between laboratories using LDTs with linearity in the range 0.363 to 0.947 and bias −0.283 to 0.359 log_10_ (Table S4).

**Fig 3:**
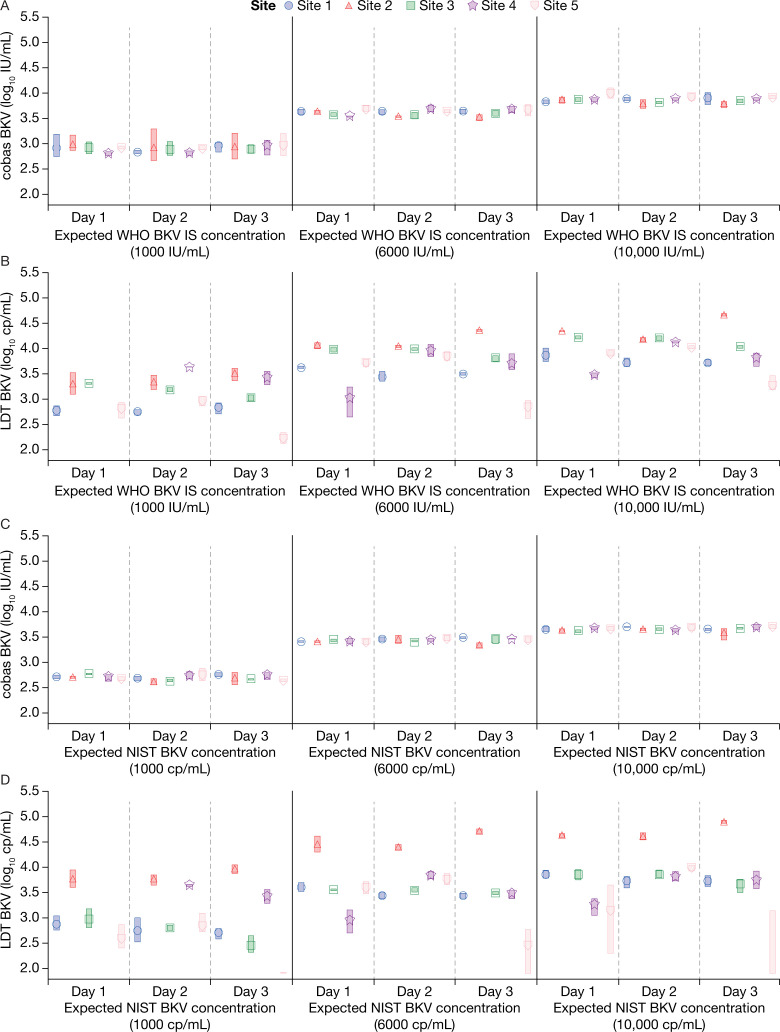
Reproducibility at each site across the three testing days at three different expected WHO BKV IS or NIST BKV standard concentrations (**A**) cobas BKV with the WHO BKV IS; (**B**) LDT BKV with the WHO BKV IS; (**C**) cobas BKV with the NIST BKV standard; (**D**) LDT BKV with the NIST BKV standard.

There was some variation across the three testing days but results from both the WHO BKV IS and NIST BKV standards were more reproducible for the cobas BKV assay compared with the LDTs (Fig. 3).

### Method comparison using surplus clinical samples

For the method comparison study using surplus clinical samples, a total of 1,045 analyzable sample pairs were tested with the cobas EBV and cobas BKV assays (544 and 501 sample pairs, respectively). Refer to Fig. S1 and S2 for the disposition of samples for EBV and BKV targets, respectively.

#### cobas EBV

**Fig 4:**
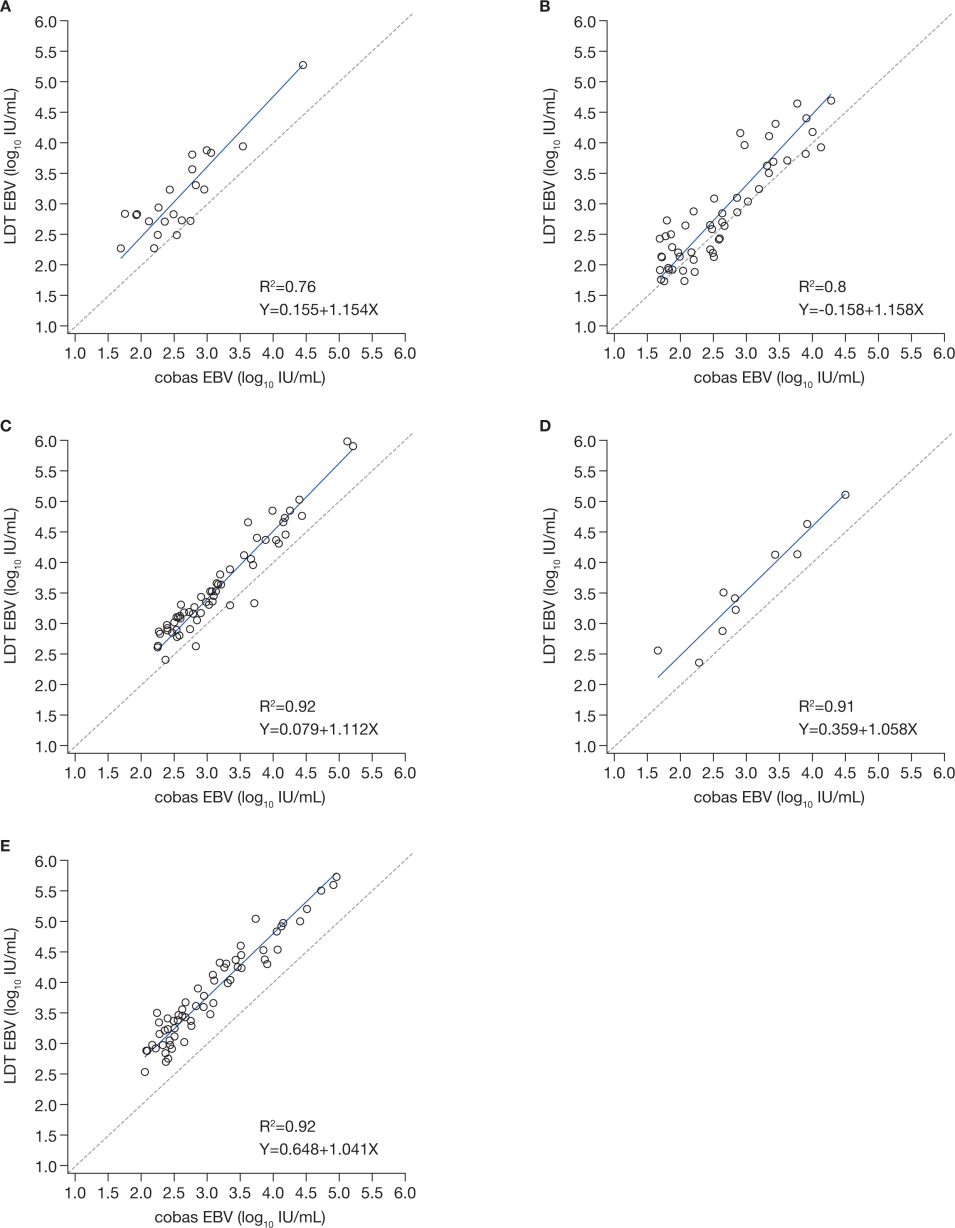
Deming linear regression analysis comparing the cobas EBV assay and the EBV LDT at each site, with LDT results converted to or given in IU/mL. (**A**) Site 1 LDT EBV vs cobas EBV (n=22); (**B**) site 2 LDT EBV vs cobas EBV (n=51); (**C**) site 3 LDT EBV vs cobas EBV (n=59); (**D**) site 4 LDT EBV vs cobas EBV (n=10);^*^ and (**E**) site 5 LDT EBV vs cobas EBV (n=61). CI, confidence interval; EBV, Epstein-Barr virus; IU, international units; LDT, laboratory-developed testing solutions; LLoQ, lower limit of quantitation. ^*^After conversion from cp to IU, only one observation was in the overlapping linear range of both the cobas EBV assay and LDT EBV, nine observations were <LLoQ for the LDT. The data shown include all 10 observations.

A total of 544 EDTA-plasma samples from five testing sites were included in the evaluation (Table S5; Fig. S1). Results reported using the existing LDT and the cobas EBV assay are shown in (Table S5): overall, the results of the EBV-positive samples at each center were consistent between the EBV LDTs and the cobas EBV assay, and the cobas EBV returned a quantitative value for a greater proportion of samples than the other assays. The EBV LDTs generally detected higher concentrations of EBV compared with the cobas EBV assay (Fig. 4; Fig. S3). There were greater differences in the quantitation of EBV by the cobas EBV and the EBV LDTs in samples with higher viral load (Fig. 4; Fig. S4).

#### cobas BKV

**Fig 5:**
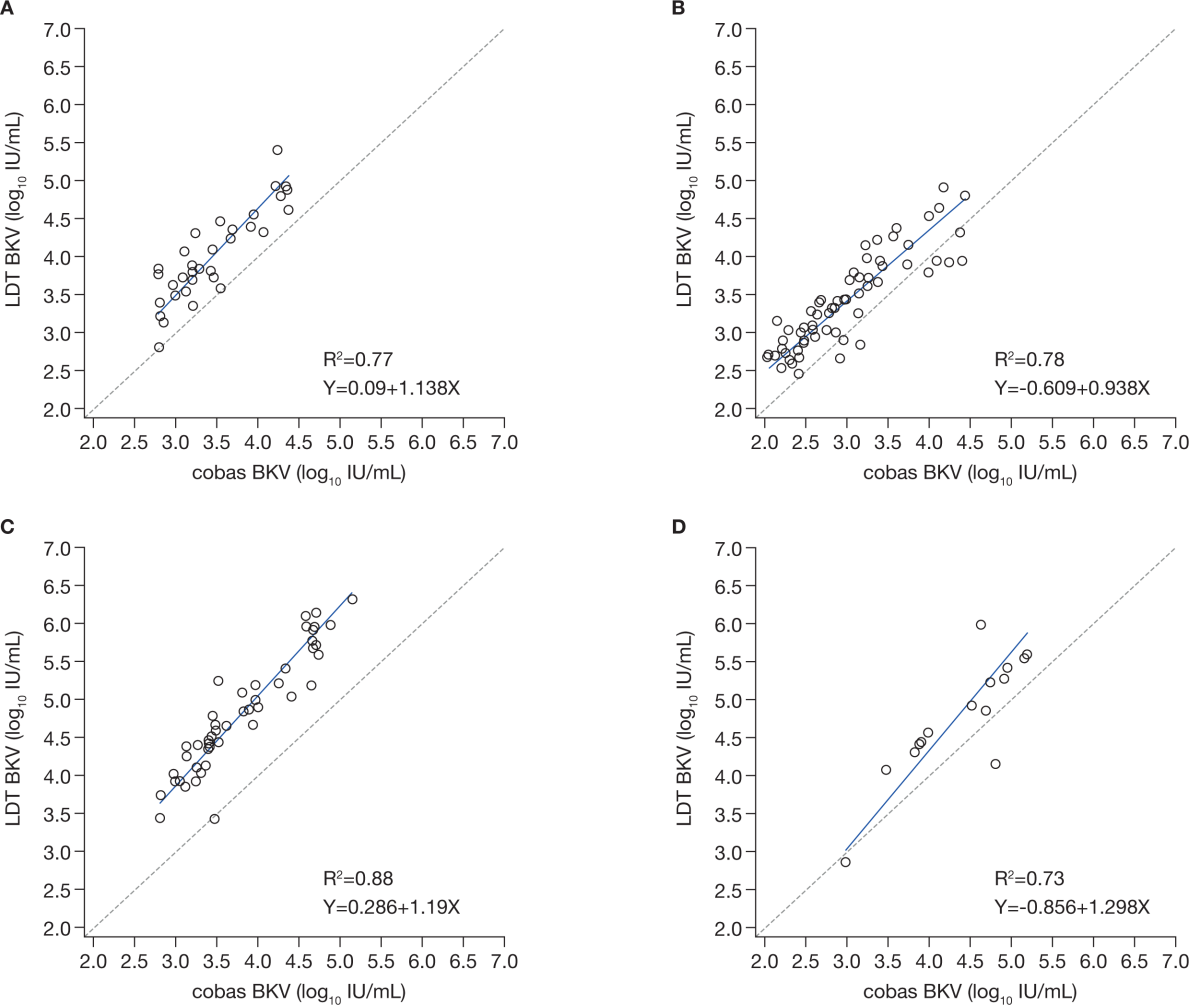
Deming linear regression analysis comparing the cobas BKV assay and the BKV LDT at each site, results converted to or given in IU/mL^*^. ^*^Data with conversion factor are not available for site 4. (**A**) Site 1 LDT BKV vs cobas BKV (n=35); (**B**) site 2 LDT BKV vs cobas BKV (n=67); (**C**) site 3 LDT BKV vs cobas BKV (n=51); (**D**) site 5 LDT BKV vs cobas BKV (n=17). BKV, BK virus; CI, confidence interval; IU, international units; LDT, laboratory-developed testing solutions.

In total, 501 samples were assessed by the cobas BKV assay and LDT (Table S6; Fig. S2): the cobas BKV returned a quantitative value for a greater proportion of samples than the other assays. For all sites, there were clear differences in the viral loads quantified by the cobas BKV and the LDT (Fig. S5). For two centers (sites 1 and 2), the values of the cobas BKV and LDT BKV were within 0.5 log IU/mL after conversion (Fig. 5; Fig. S6).

## DISCUSSION

Monitoring of both EBV and BKV DNA is a key aspect of the management of transplant patients, but assay variability in measuring both EBV and BKV DNA complicates the interpretation of results and the establishment of thresholds for clinical management decision-making (8, 18). The variability between LDTs for the detection of EBV and predicting EBV-associated PTLD is well known, with many centers developing their in-house cut-off values for normal EBV (8). Ideally, DNA levels indicating the risk of EBV-associated PTLD following transplant would be standardized and commutable between centers (4, 26) and patient populations. Guidelines recommend that renal transplant and HSCT patients’ viral load should be assessed regularly in the first 1–2 years post-transplant for the detection of increased risk of complications such as PTLD or BK virus-associated nephritis (9, 13, 15). EBV and BKV DNA levels are used to guide treatment decisions, and/or adjustment of immunosuppression, and inform histopathologic studies, which can have a significant impact on patient management (9, 13, 15). Thus, the accuracy and reproducibility of quantitative assays are critical.

The purpose of this study was to evaluate the performance of the cobas EBV and cobas BKV assays across five laboratories and compare the results against LDTs; subsequently, we also gained insights into the comparability and performance of these laboratory solutions currently used throughout Europe. The data from this study confirm the greater interlaboratory variability for both EBV and BKV LDTs in comparison to assays that are standardized to the WHO IS (such as the cobas assays and site 5’s EBV LDT), in agreement with the outcomes of previous studies (27–30). For example, of the five EBV LDTs tested, three consistently estimated much higher viral loads relative to the WHO EBV IS, with site 1 returning a value almost 1.0 log higher than expected.

The cobas EBV and cobas BKV assays reported quantitative values for a greater proportion of samples than the LDTs, which can be expected given the cobas EBV and cobas BKV assays had the widest linear ranges and lowest lower limit of quantitation of all the assays under evaluation (reported in Tables 1 and 2).

Our study also suggests that there may be additional variability for both EBV and BKV LDTs between testing days at some centers, with variability such that it confounds differentiation of the 1,000, 6,000, and 10,000 IU/mL panels in some cases. This was observed both when considering the scope of interlaboratory testing, as was noted early as a challenge of laboratory-specific solutions but also with intralaboratory comparisons, despite using the same assay. This uncertainty of measurement may impact the trending of patient results over time. In contrast to the LDTs, the cobas EBV and cobas BKV assays provided consistent agreement with their respective international standards between testing sites and different testing days, demonstrating minimal variation, and good accuracy and reproducibility. It is worth noting that a single isolate may not take into consideration sequence differences of clinical variants, viral genome integrity, or nucleic acid fragmentation that can be encountered when processing clinical specimens. Our study is limited to show bias discrepancies with contrived panels only and against individual assays using local clinical sample sets.

The purpose of the international standards is to allow harmonization of viral load data in clinical samples. Comparison of the cobas EBV and cobas BKV assays with the LDTs using “real-world” clinical samples revealed that, once the LDTs were adjusted using the WHO IS, their results were generally more closely aligned with the cobas EBV and cobas BKV assays. However, the results reported by the LDT from site 5, which was developed using the WHO IS, still had some variance vs the expected concentrations. Although the LDT results for all sites were not entirely commutable even once converted using the reference standard, differences between two assays were greatest at higher and lower viral loads, indicating proportional bias, likely due to loss of linearity with the LDTs near the limits of quantitation in relation to the cobas assays. This may also be explained in part by a study limitations related to both the limited number of repeats that were possible using the WHO IS used to develop the conversion factor from cp/mL to IU/mL for each LDT and the fact that some sites were unable to reach the target number of clinical samples, as detailed in the methods. It is of note that there may be differences in the commutability of the WHO standard materials between assays. For EBV, these differences may be related to viral fragmentation in routine clinical samples vs the standard material (31); while no assays in this study target BamHI, it is worth noting that some assays utilize this target with repeat copies to increase sensitivity, but poses a confounding effect when utilized for quantitation (32). For BKV, these differences may be due to deletions in the BKV nucleotide sequence in clinical samples in the regions targeted by LDTs (33). The WHO BKV IS contains sub-populations with various deletions in the large T antigen region (33), and thus using this standard to calibrate an assay that targets this region of the virus can lead to inaccurate results. The LDT for site 3 targets this region and may account for some of the offset compared to cobas, as the LDT may over quantitate clinical samples not carrying the deletion. In support of this limitation, the LDTs for BKV performed more consistently when using the NIST BKV standard than with the WHO BKV IS. Alongside the choice of assay, it is therefore important to consider which standard to utilize when deciding upon a testing methodology. When considering the use of either the NIST standard or WHO IS, it is important to highlight the potential challenge of the WHO IS having large T antigen deletions, which may impact calibration for tests targeting this region. Variability was still observed using the NIST standard; however, this was marginal compared to the variability observed using the LDTs. The authors do not make the recommendation to use one or the other, but laboratories should be aware of the difference in the standards, and the use of standardized assays should improve our future understanding of the relevance of these differences.

The majority of quantitative testing for transplant-associated viruses such as EBV and BKV is performed using LDTs with a range of different genetic targets, extraction and amplification methods, and testing platforms (19).

Current guidelines recommend that patients should be monitored for viral load by the same assay in a single laboratory, with a need for cross-referencing of results if interlaboratory comparisons are needed (5), which has the potential to complicate management. More standardized solutions may help to mitigate this need, although studies suggest that variance still persists (19, 34): a recent publication from the United States reported an approximate 0.5 log_10_ IU/mL offset between the cobas EBV and cobas BKV assays and an LDT, despite the assays reporting in IU/mL (34).

In conclusion, in the present study, the cobas EBV and cobas BKV assays provided a greater degree of accuracy vs the WHO IS, which suggests that, apart from more consistent and reliable data, these assays may also provide greater clinical certainty around changes in viral load during post-transplant monitoring. The high degree of interlaboratory reproducibility seen with the cobas EBV and cobas BKV assays allows for comparability of results, which will increase understanding of the implications of viral loads and potential threshold values. It would seem that the body of evidence to establish guidance thresholds is more likely to develop after more laboratories transition to systems with demonstrated inter-site commutability. It is an important consideration that clinical thresholds established using a standardized IVD system might not be directly transferrable to LDTs on other systems. Future work is needed in this area; however, it is worth noting that in the absence of this particular commercial solution, data in this study indicate that standardization with LDTs in general can help mitigate some of these issues. Ultimately, as treatment for EBV and BKV are driven by the viral load test result, the more accurate and comparable the test result is across institutions, the more informed and better treatment decisions can be made. Errors in viral load quantification can lead to inappropriate clinical decision-making, such as failure to diagnose infection, unnecessary allograft biopsy, and premature reduction in immunosuppression (5, 15, 17).

## SUPPLEMENTAL MATERIAL

Supplemental tables and figures jcm.00267-24-s0001.docx Details of EBV and BKV LDTs at each testing site and further data on final sample sizes, linearity and accuracy of the EBV and BKV assays for viral DNA quantitation using the WHO BKV IS or NIST BKV standard, assay reproducibility, etc.

ASM does not own the copyrights to Supplemental Material that may be linked to, or accessed through, an article. The authors have granted ASM a non-exclusive, world-wide license to publish the Supplemental Material files. Please contact the corresponding author directly for reuse.
