# Supplementary material for: Performance of the cobas EBV and cobas BKV assays: multi-site comparison of standardized quantitation
Source: J Clin Microbiol. 2024 Jul 24;62(8):e00267-24. doi: 10.1128/jcm.00267-24 (PMC11323559; doi:10.1128/jcm.00267-24)
Supplement: Supplemental tables and figures — Details of EBV and BKV LDTs at each testing site and further data on final sample sizes, linearity and accuracy of the EBV and BKV assays for viral DNA quantitation using the WHO BKV IS or NIST BKV standard, assay reproducibility, etc. [file jcm.00267-24-s0001.docx]

**Performance of the cobas EBV and cobas BKV assays: multi-site comparison of standardized quantitation**

Laura Mannonen,^1^ Pia Jokela,^1^ Marianne Kragh Thomsen,^2^ Sabine Yerly,^3^ Gustavo Cilla,^4^ Daniel Jarem,^5^ Jesse A Canchola,^6^ Mark Hopkins^7,8,#^

^1^Department of Clinical Microbiology, HUS Diagnostic Center, HUSLAB, Clinical Microbiology, University of Helsinki and Helsinki University Hospital, Topeliuksenkatu 32, 00290 Helsinki, Finland
^2^Department of Clinical Microbiology, Aarhus University Hospital, Palle Juul-Jensens Boulevard 99, DK-8200 Aarhus N, Denmark
^3^Laboratory of Virology, Diagnostic Department, Geneva University Hospitals, Geneva, Switzerland
^4^Department of Microbiology, Donostia University Hospital and Biodonostia Health Research Institute, San Sebastián, Spain ^5^Clinical Development and Medical Affairs, Roche Molecular Systems, Inc., 4300 Hacienda sDrive, Pleasanton, CA, USA
^6^CDMA Biometrics, Biostatistics Group, Roche Molecular Systems, Inc., 4300 Hacienda Drive, Pleasanton, CA, USA

^7^Virology, Barts Health NHS Trust, London, UK

^8^Department of Infection and Immunity, Liverpool University Hospitals NHS Trust, Liverpool, UK

# Table S1. Final sample sizes for analytical performance and method correlation studies

| **Site** | **Analytical performance** | | **Method correlation** | |
| --- | --- | --- | --- | --- |
|  | **EBV** | **BKV** | **EBV** | **BKV** |
| **1** | 36 | 63 | 71 | 63 |
| **2** | 36 | 63 | 139 | 140 |
| **3** | 36 | 63 | 139 | 140 |
| **4** | 36 | 63 | 55 | 81 |
| **5** | 36 | 63 | 140 | 77 |
| **Total** | 180 | 315 | 544 | 501 |
| **Group total** | 495 | | 1045 | |
| **Grand total** | 1540 | | | |

BKV, BK virus; EBV, EBV, Epstein-Barr virus.

# Table S2. Final sample sizes for reproducibility at each site across the three testing days at three different expected WHO BKV IS or NIST BKV standard concentrations

| **Site** | **Day 1** | **Day 2** | **Day 3** | **Total** |
| --- | --- | --- | --- | --- |
| **1** | 9 | 9 | 9 | 27 |
| **2** | 9 | 9 | 9 | 27 |
| **3** | 9 | 9 | 9 | 27 |
| **4** | 9 | 9 | 9 | 27 |
| **5** | 9 | 9 | 9 | 27 |
| **Total** | 45 | 45 | 45 | 135 |

NIST BKV, National Institute of Standards and Technology DNA quantitative standard for BKV; WHO BKV IS, WHO international standard for BKV.

# Table S3. Linearity and accuracy of the cobas EBV assay and the LDT for EBV DNA quantitation at each site using the WHO EBV IS

|  | **cobas EBV** | | **LDT EBV** | |
| --- | --- | --- | --- | --- |
| **Site** | Linearity R^2^ | Bias: Mean observed difference (log_10_) | Linearity R^2^ | Bias: Mean observed difference (log_10_)^*^ |
| **1** | 0.992 | −0.023 | 0.979 | 0.914 |
| **2** | 0.970 | −0.112 | 0.856 | 0.724 |
| **3** | 0.984 | −0.095 | 0.932 | 0.470 |
| **4** | 0.988 | −0.068 | 0.865 | −0.612 |
| **5** | 0.992 | −0.083 | 0.476 | −0.053 |

^*^Average accuracy/bias = log_10_ linearized quant − log_10_ target concentration, where the log_10_ linearized quant is the predicted value from a regression equation fit on the data points in the dilution series with slope of 1.0.

EBV, Epstein-Barr virus; LDT, laboratory-developed testing solutions; WHO EBV IS, WHO international standard for EBV.

# Table S4. Linearity and accuracy of the cobas BKV assay and the LDT for BKV DNA quantitation at each site using the WHO BKV IS or NIST BKV standard

|  | **cobas BKV** | | **LDT BKV** | |
| --- | --- | --- | --- | --- |
| **Site** | Linearity R^2^ | Bias: mean observed log difference (log_10_) | Linearity R^2^ | Bias: mean observed log difference (log_10_)* |
| **WHO BKV IS** | | | | |
| **1** | 0.955 | −0.126 | 0.917 | −0.248 |
| **2** | 0.878 | −0.154 | 0.871 | 0.755 |
| **3** | 0.971 | −0.150 | 0.882 | −0.237 |
| **4** | 0.970 | −0.139 | 0.178 | −0.254 |
| **5** | 0.960 | −0.074 | 0.070 | −0.747 |
| **NIST BKV** | | | | |
| **1** | 0.990 | −0.311 | 0.947 | −0.244 |
| **2** | 0.973 | −0.362 | 0.884 | 0.359 |
| **3** | 0.987 | −0.332 | 0.946 | 0.153 |
| **4** | 0.986 | −0.307 | 0.363 | −0.251 |
| **5** | 0.980 | −0.320 | 0.543 | −0.283 |

*Average accuracy = log_10_ linearized quant – log_10_ target concentration, where the log_10_ linearized quant is the predicted value from a regression equation fit on the data points in the dilution series with slope of 1.0.

BKV, BK virus; LDT, laboratory-developed testing solutions; NIST BKV, National Institute of Standards and Technology DNA quantitative standard for BKV; WHO BKV IS, WHO international standard for BKV.

# Table S5. The performance of the cobas EBV assay compared with the overall performance of the LDTs for EBV

|  | **cobas EBV assay^*^** | | | |
| --- | --- | --- | --- | --- |
|  | **Target not detected** | **< lower limit of quantification** | **Within the linear range** | **Total** |
| **LDT site 1**^†^ |  |  |  |  |
| Target not detected | 17 | 0 | 2 | 19 |
| < Lower limit of quantification | 12 | 0 | 3 | 15 |
| Within the linear range of LDT | 3 | 0 | 34 (22^‡^) | 37 |
| Total | 32 | 0 | 39 | 71 |
| **LDT site 2**^†^ |  | | | |
| Target not detected | 56 | 0 | 4 | 60 |
| < Lower limit of quantification | 1 | 0 | 1 | 2 |
| Within the linear range of LDT | 2 | 0 | 75 (51^‡^) | 77 |
| Total | 59 | 0 | 80 | 139 |
| **LDT site 3**^†^ |  | | | |
| Target not detected | 28 | 0 | 12 | 40 |
| < Lower limit of quantification | 3 | 0 | 17 | 20 |
| Within the linear range of LDT | 0 | 0 | 79 (59^‡^)^§^ | 79 |
| Total | 31 | 0 | 108 | 139 |
| **LDT site 4**^†^ |  | | | |
| Target not detected | 39 | 0 | 6 | 45 |
| < Lower limit of quantification | 0 | 0 | 9 | 9 |
| Within the linear range of LDT | 0 | 0 | 1 | 1 |
| Total | 39 | 0 | 16 | 55 |
| **LDT site 5**^†^ |  | | | |
| Target not detected | 39 | 0 | 8 | 47 |
| < Lower limit of quantification | 5 | 0 | 11 | 16 |
| Within the linear range of LDT | 0 | 0 | 77 (61^‡^) | 77 |
| Total | 44 | 0 | 96 | 140 |

^*^Linear range for cobas EBV assay: 1.40E+01 IU/mL to 2.30E+08 IU/mL.

^†^Linear range for the EBV LDTs: site 1) 37.92 IU/mL to 1.2E6 IU/mL; site 2) 47.5 IU/mL to 2.96E5 IU/mL; site 3) 170 IU/mL to 1.7E6 IU/mL; site 4) 4.62E4 IU/mL to 4.62E8 IU/mL; site 5) 100 IU/mL to 1E6 IU/mL.

^‡^Samples with results reported by both assays within their overlapping linear ranges.

^§^For one sample for both the LDT and the cobas EBV assay, viral load was greater than the upper limit of quantification.

EBV, Epstein-Barr virus; IU, international units; LDT, laboratory-developed testing solutions.

# Table S6. The performance of the cobas BKV assay compared with the overall performance of the LDTs for BKV

|  | **cobas BKV assay**^*^ | | | |
| --- | --- | --- | --- | --- |
|  | **Target not detected** | **< lower limit of quantification** | **Within the linear range** | **Total** |
| **LDT site 1**^†^ |  |  |  |  |
| Target not detected | 15 | 0 | 5 | 20 |
| < Lower limit of quantification | 0 | 0 | 1 | 1 |
| Within the linear range of LDT | 0 | 0 | 42 (35^‡^) | 42 |
| Total | 15 | 0 | 48 | 63 |
| **LDT site 2**^†^ |  | | | |
| Target not detected | 37 | 0 | 3 | 40 |
| < Lower limit of quantification | 3 | 0 | 17 | 20 |
| Within the linear range of LDT | 0 | 0 | 80 (67^‡^) | 80 |
| Total | 40 | 0 | 100 | 140 |
| **LDT site 3**^†^ |  | | | |
| Target not detected | 32 | 0 | 8 | 40 |
| < Lower limit of quantification | 0 | 0 | 20 | 20 |
| Within the linear range of LDT | 0 | 0 | 80 (51^‡^) | 80 |
| Total | 32 | 0 | 108 | 140 |
| **LDT site 4**^†^ |  | | | |
| Target not detected | 54 | 0 | 9 | 63 |
| < Lower limit of quantification | 1 | 0 | 16 | 17 |
| Within the linear range of LDT | 0 | 0 | 1 | 1 |
| Total | 55 | 0 | 26 | 81 |
| **LDT site 5**^†^ |  | | | |
| Target not detected | 36 | 0 | 5 | 41 |
| < Lower limit of quantification | 0 | 0 | 16 | 16 |
| Within the linear range of LDT | 0 | 0 | 20 (17^‡^) | 20 |
| Total | 36 | 0 | 41 | 77 |

^*^Linear range for the cobas BKV assay: 7.41E+01 IU/mL to 7.41E+08 IU/mL.

^†^Linear range for the BKV LDTs: site 1) 569 IU/mL to 21.6E8 IU/mL; site 2) 100 IU/mL to 2E5 IU/mL; site 3) 640 IU/mL to 6.4E9; site 4) 1.1E4 cp/mL to 1.1E8 cp/mL; site 5) 6.3E2 IU/mL to 2.1E11 IU/mL.

^‡^Samples with results reported by both assays within their overlapping linear ranges.

BKV, BK virus; cp, copies; IU, international units; LDT, laboratory-developed testing solutions.

Figure S1. LDT EBV Sample disposition flow chart


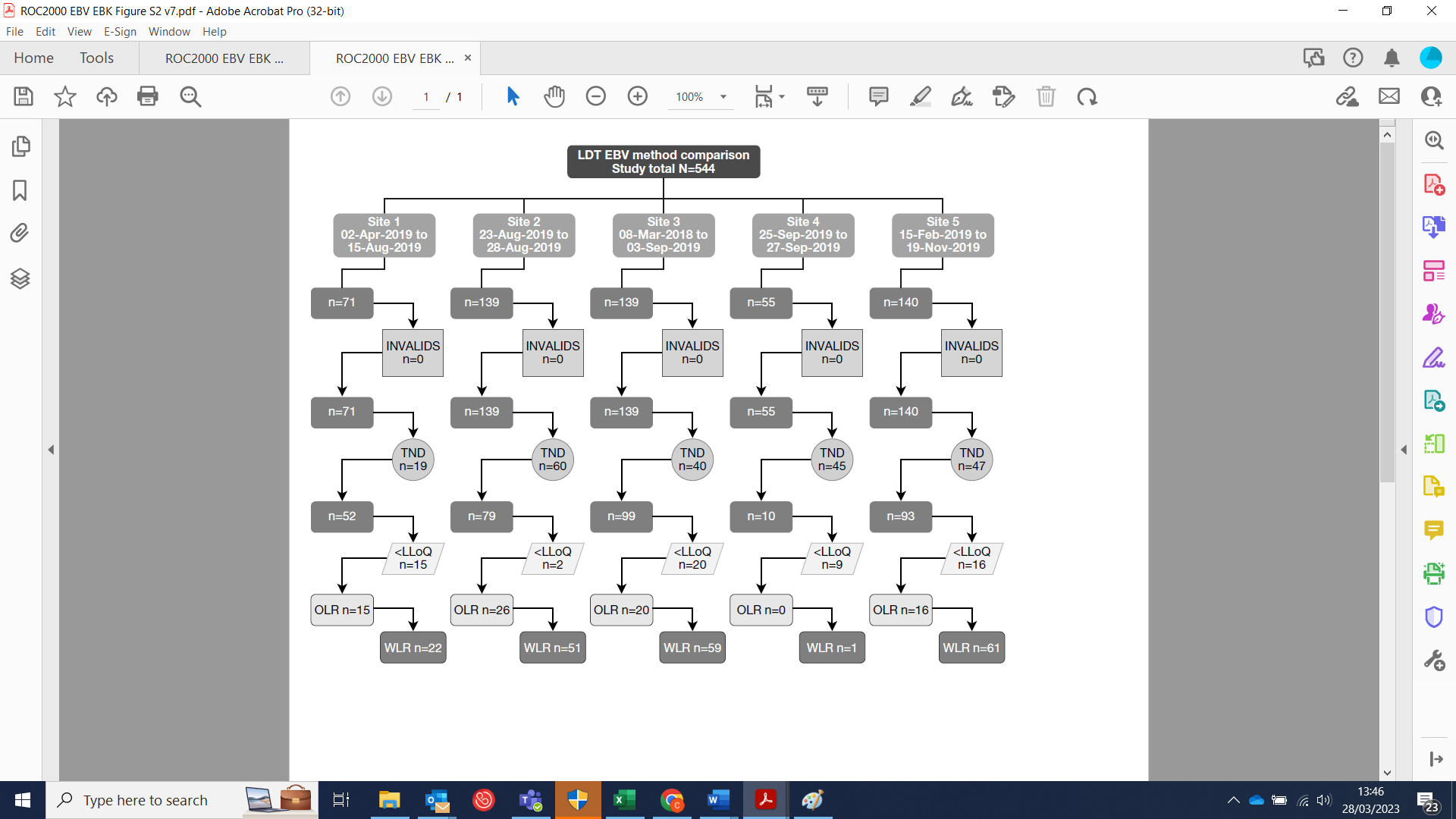


EBV, Epstein-Barr virus; LDT, laboratory-developed test; LLoQ, lower limit of quantification; OLR, outside the linear range of the cobas EBV assay; TND, target not detected; WLR, within the overlapping linear range of both assays.

# Figure S2. LDT BKV sample disposition flow chart


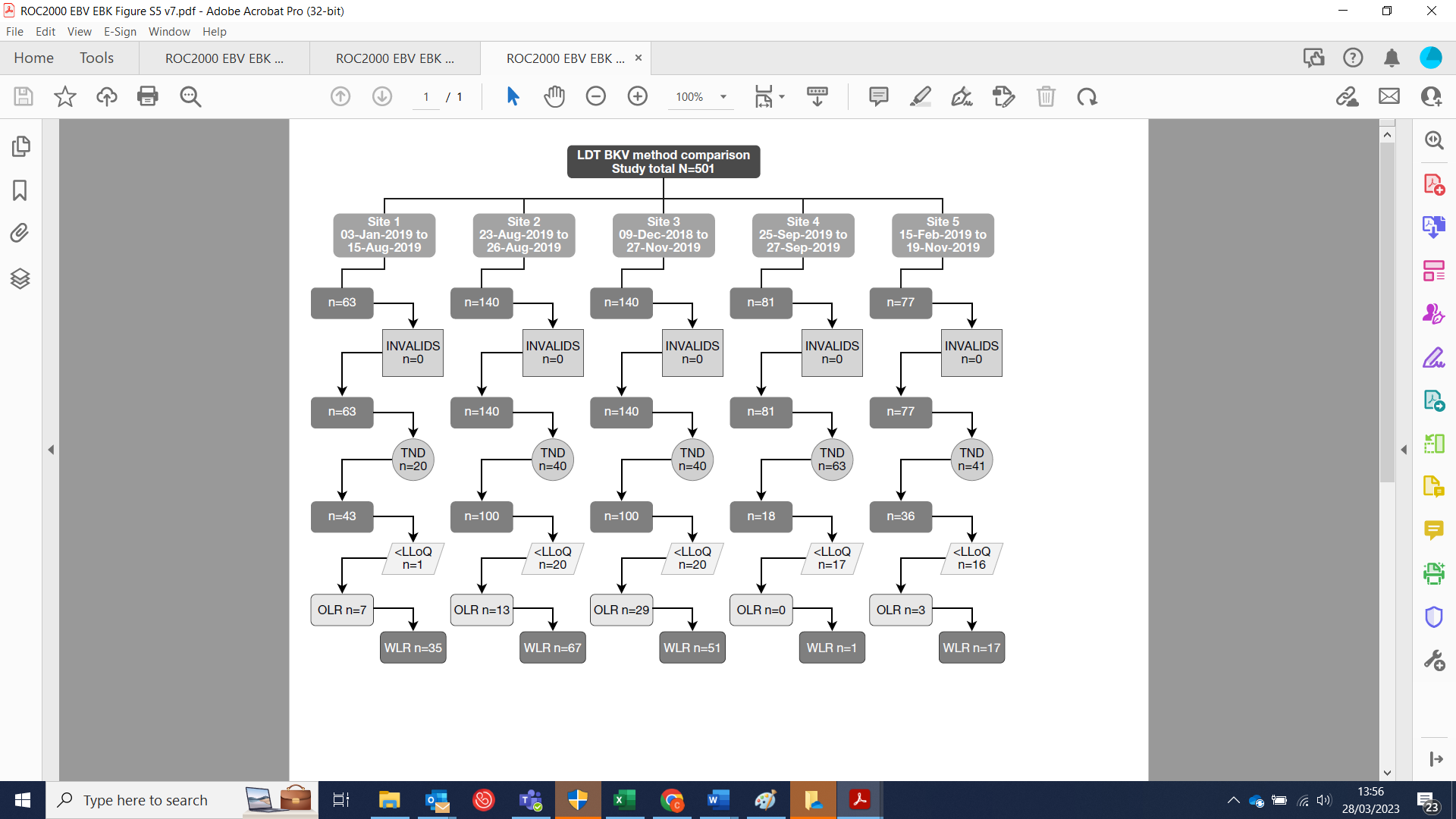


BKV, BK virus; LDT, laboratory-developed test; LLoQ, lower limit of quantification; OLR, outside the linear range of the cobas EBV assay; TND, target not detected; WLR, within the overlapping linear range of both assays.

# Figure S3. Deming linear regression analysis comparing the cobas EBV assay and the LDT EBV at each site, without conversion*

^*^Although there were originally 10 observation pairs for site 4, after conversion from cp to IU, the overlapping range includes only one observation pair.

**A**: site 1 LDT EBV vs cobas EBV (n=22); **B**: site 2 LDT EBV vs cobas EBV (n=51);

**C**: site 3 LDT EBV vs cobas EBV (n=59); **D**: site 4 LDT EBV vs cobas EBV (n=10);^*^

**E**: site 5 LDT EBV vs cobas EBV (n=61).


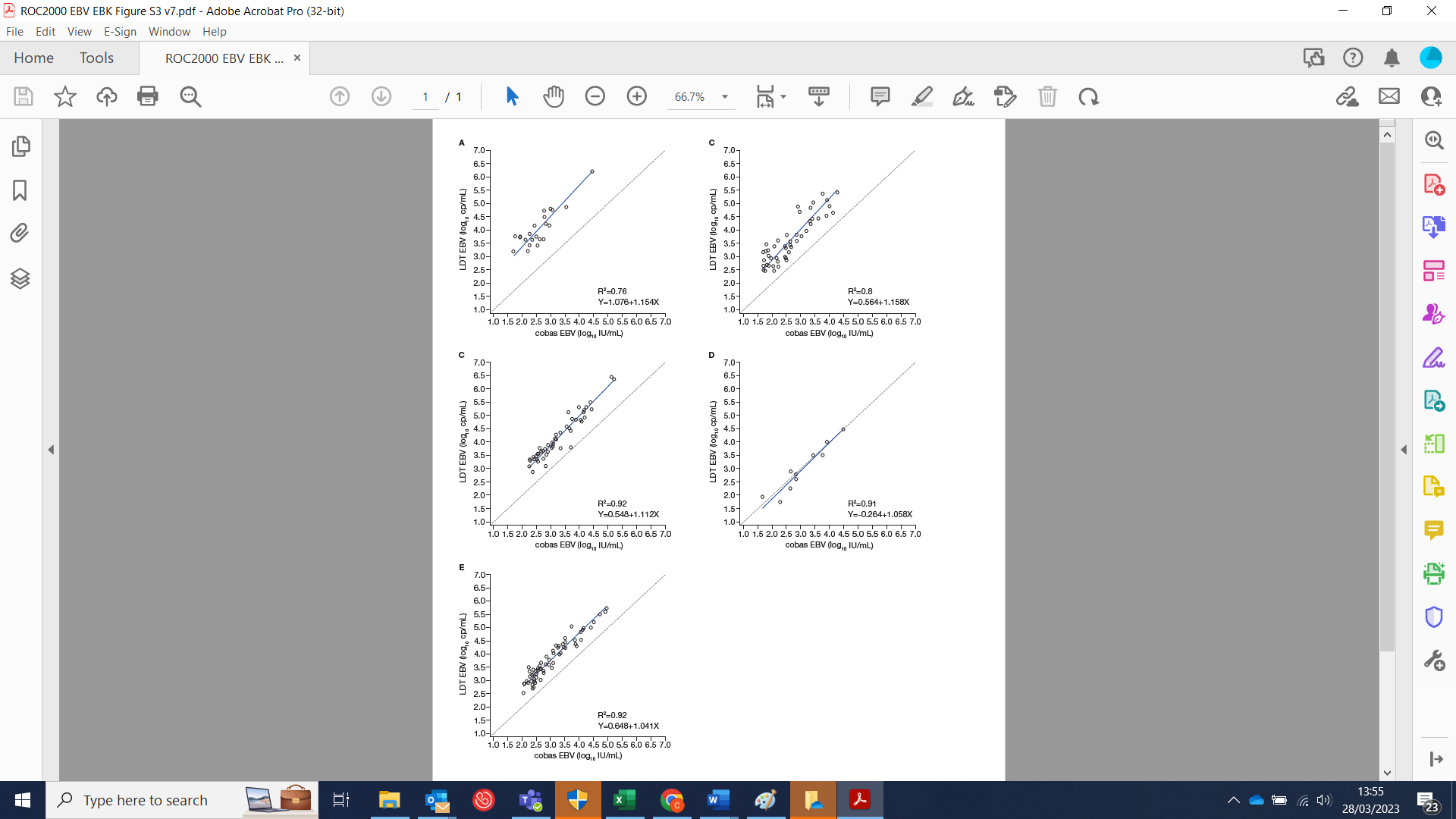


CI, confidence interval; cp, copies; EBV, Epstein-Barr virus; IU, international units;
LDT, laboratory-developed testing solutions.

# Figure S4. Bland–Altman analysis comparing the cobas EBV assay and the EBV LDT at each site^*^

Data presented with the classical least squares regression line. Results reported are those within both assays’ overlapping linear ranges. The 95% lower and upper limits of agreement are represented by dotted lines; the bias is represented by a dashed line. ^*^Data with conversion factor are not available for site 4. Therefore, the graph for this site is not shown.

**A**: site 1 LDT EBV vs cobas EBV (n=22; bias [mean difference]=0.551; lower and upper limit of agreement=-0.127, 1.228);

**B**: site 2 LDT EBV vs cobas EBV (n=51; bias [mean difference]=0.251; lower and upper limit of agreement=-0.494, 0.996);

**C**: site 3 LDT EBV vs cobas EBV (n=59; bias [mean difference]=0.436; lower and upper limit of agreement=-0.017, 0.889);

**D**: site 5 LDT EBV vs cobas EBV (n=61; bias [mean difference]=0.773; lower and upper limit of agreement=0.338, 1.207).


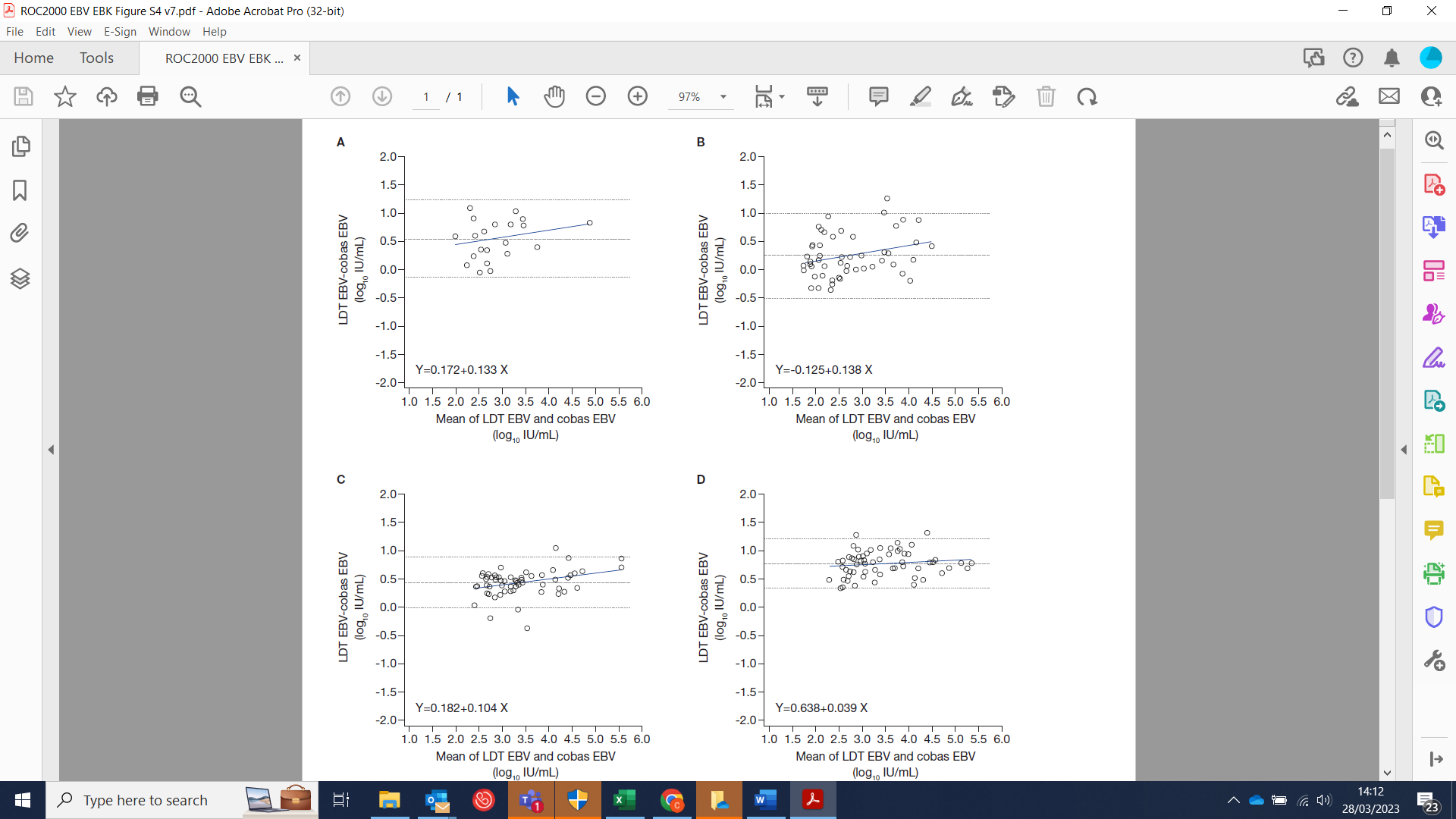


CI, confidence interval; diff, difference; EBV, Epstein-Barr virus; IU, international units; LDT, laboratory-developed testing solutions; LL, lower limit; UL, upper limit.

# Figure S5. Bland–Altman analysis comparing the cobas BKV assay and the LDT BKV at each site^*^

Data presented with the ordinary least squares regression line. Results reported are those within both assays’ overlapping linear ranges. The 95% lower and upper limits of agreement are represented by dotted lines; the bias is represented by a dashed line. ^*^Site 4 originally had 17 observation pairs. However, after conversion from cp to IU, there was only one observation pair in the overlapping linear range of both assays. Therefore, the graph for this site is not shown.

**A**: site 1 LDT BKV vs cobas BKV (n=35; bias [mean difference]=0.566; lower and upper limit of agreement=0.022, 1.11);

**B**: site 2 LDT BKV vs cobas BKV (n=67; bias [mean difference]=0.423; lower and upper limit of agreement=-0.172, 1.019);

**C**: site 3 LDT BKV vs cobas BKV (n=51; bias [mean difference]=1.005; lower and upper limit of agreement=0.465, 1.544);

**D**: site 5 LDT BKV vs cobas BKV (n=17; bias [mean difference]=0.454; lower and upper limit of agreement=-0.383, 1.291).


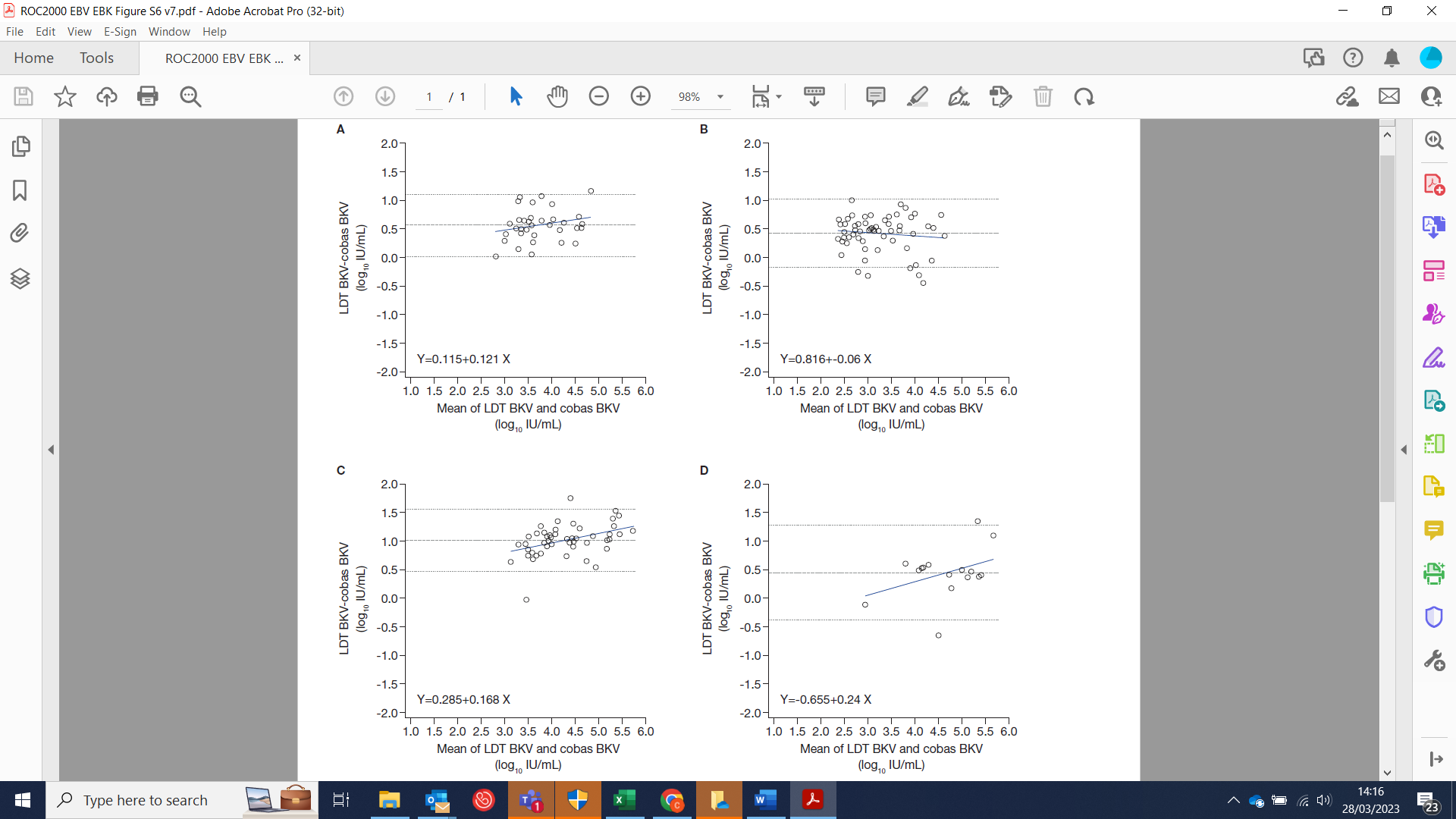


BKV, BK virus; CI, confidence interval; cp, copies; diff; difference; IU, international units; LDT, laboratory-developed testing solutions; LL, lower limit; UL, upper limit.

# Figure S6. Deming linear regression analysis comparing the cobas BKV assay and the BKV LDT at each site, without conversion^*^

^*^After conversion from cp to IU, only one observation was in the overlapping linear range of both the cobas EBV assay and LDT EBV, six observations were <LLoQ for the LDT. Data shown includes all 17 observations.

**A**: site 1 LDT BKV vs cobas BKV (n=35); **B**: site 2 LDT BKV vs cobas BKV (n=67);

**C**: site 3 LDT BKV vs cobas BKV (n=51); **D**: site 4 LDT BKV vs cobas BKV (n=17);^*^

**E**: site 5 LDT BKV vs cobas BKV (n=17).


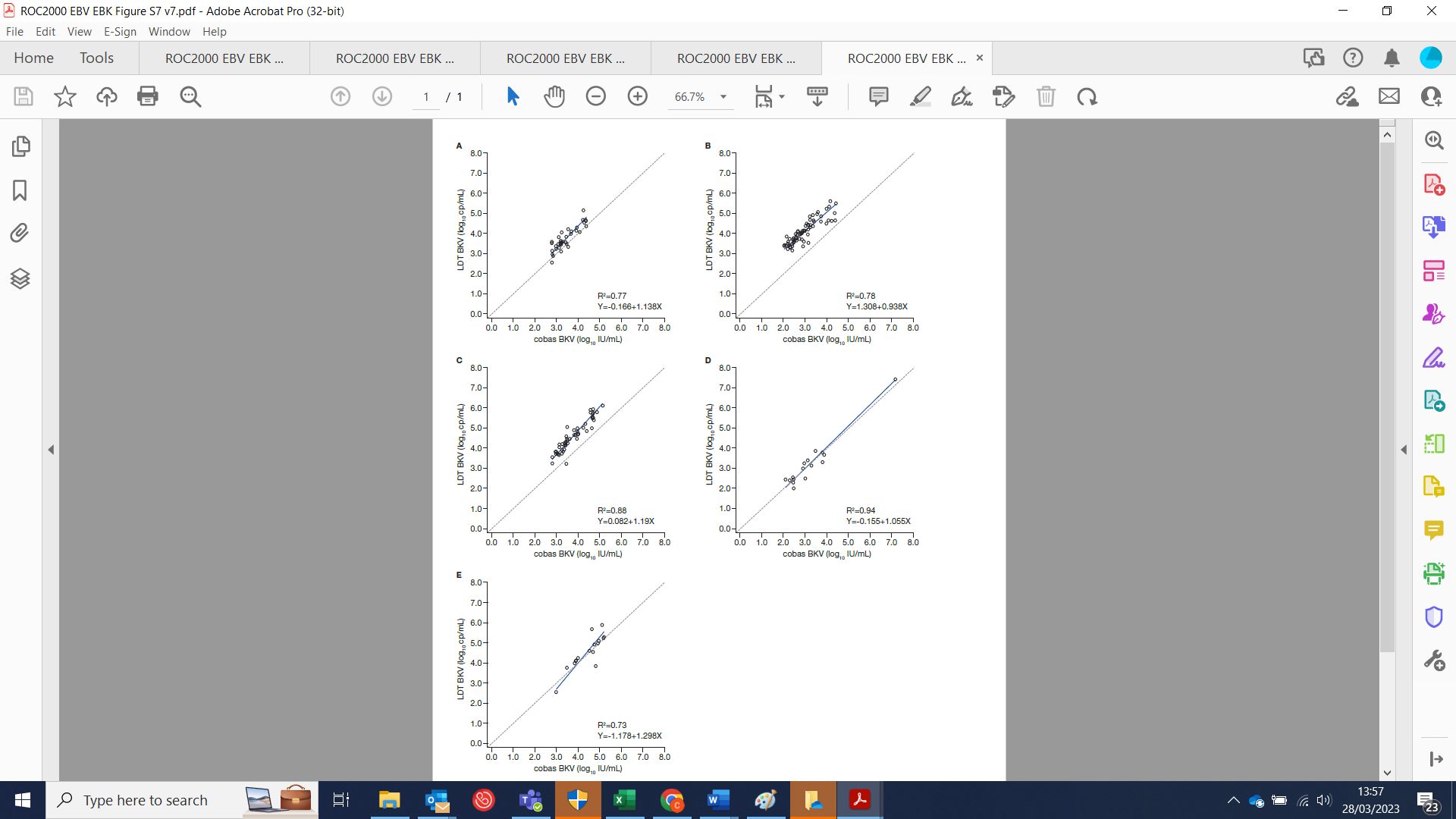


BKV, BK virus; CI, confidence interval; cp, copies; IU, international units; LDT, laboratory-developed testing solutions; LLoQ, lower limit of quantification.
